# 

**Published:** 1870-03

**Authors:** 


### Editorial Department.

### American Medical Association.

The twenty-first Annual Session will be held in Washington, D. C., May 3, 1870, at 11 A. M. The following Committees are expected to report:

On Cultivation of the Cinchona Tree.—Dr. Lemuel J. Deal, Pennsylvania, Chairman.

On the Cryptogamic Origin of Disease with special reference to recent microscopic investigations on that subject,—Dr. Edward Curtis, U. S. A., Chairman.

On the Doctrine of Force, Physical and Vital.—Dr. John Waters, Missouri, Chairman.

On Variola.—Dr. Joseph Jones, Louisiana, Chairman.

On the Relative Advantages of Syme's and Pirogroff's mode of Amputating at the Ankle.—Dr. G. A. Otis, U. S. A., Chairman.

On a National Medical School.—Dr. F. G. Smith, Pennsylvania, Chairman.

On Commissioners to aid in Trials involving Scientific Testimony.—Dr. John Ordronaux, N. Y., Chairman.

On the Climatology and Epidemics of—Maine, Dr. J. C. Weston; New Hampshire, Dr. P. A. Stackpole; Vermont, Dr. Henry Janes; Massachusetts, Dr. H. I. Bowditch; Rhode Island, Dr. C. W. Parsons; Connecticut, Dr. E. K. Hunt; New York, Dr. W. F. Thomas; New Jersey, Dr. Ezera M. Hunt; Pennsylvania, Dr. D. F. Condie; Maryland, Dr. O. S. Mahon; Georgia, Dr. Juriah Harris; Missouri, Dr. George Englemann; Alabama, Dr. R. F. Michel; Texas, Dr. T. J. Heard; Illinois, Dr. R. C. Hamil; Indiana, Dr. J. F. Hibberd; District of Columbia, Dr. T. Antisel; Iowa, Dr. J. C. Hughes; Michigan, Dr. Abm. Sager; Ohio, Dr. T. L. Neal; California, Dr. F. W. Hatch; Tennessee, Dr. B. W. Avent; West Virginia. Dr. E. A. Hildebreth; Minnesota, Dr. Samuel Willey; Virginia, Dr. W. O. Owen; Delaware, Dr. L. B. Bush; Arkansas, Dr. G. W. Lawrence; Mississippi, Dr. W. Compton; Louisiana, Dr. L. T. Pimm; Wisconsin, Dr. J. K. Bartlett; Kentucky, Dr. J. D. Jackson,

On Veterinary Colleges.—Dr Thomas Antisell, D. C. Chairman.

On Medical Ethics.—Dr. Lewis A Sayre, N. Y., Chairman.

On American Medical Necrology.—Dr. C. C. Cox, Maryland, Chairman.

To Memoralize State Medical Societies.—Dr. N. S. Davis, Illinois, Chairman.

On Nomenclature of Diseases.—Dr. F. G. Smith, Chairman.

On Medical Education.—Dr. T. G, Richardson, Louisiana Chairman.

On Medical Literature.—Dr. J. J. Woodward, U. S. A., Chairman.

On Prize Essays.—Dr. Grafton Tyler, D. C., Chairman.

Voluntary communications will be presented by—Dr. John Curwen, Pennsylvania.—On the Proper Treatment of the Insane.

Dr. Nathan Allen, Massachusetts.—On the Physiological Laws of Human Increase.

Secretaries of all organizations are requested to forward lists of their Delegates as soon as elected, to the Permanent Secretary.

Any respectable physician who may desire to attend, but cannot do so as a delegate, may be made a *member by invitation*, upon the recommendation of the Committee of Arrangements.

W. B. ATKINSON.

### Books and Pamphlets Received.

A Practical Treatise on the Diseases of Children. By J. Forsyth Meigs, M. D., one of the Physicians to the Pennsylvania Hospital; Consulting Physician to the Children's Hospital, etc.; and William Pepper, M. D., one of the Physicians to the Philadelphia Hospital. Lecturer on Morbid Anatomy at the University of Pennsylvania, etc. Fourth edition [Meigs on children] Revised and enlarged. Philadelphia, Lindsay and Blakiston. Received through T. Butler & Son.

A Practical Guide to the study of the Diseases of the Eye. Their Medical and Surgical Treatment. By Henry W. Williams, A. M., M. D., Ophthalmic Surgeon to the City Hospital, Boston; University Lecturer on Ophthalmic Surgery in Harvard University; President of the American Ophthalmological Society, etc. Third Edition revised and enlarged. Boston: Fields, Osgood & Co.

The Physical Life of Woman. Advice to the Maiden, Wife and Mother. By George Napheys, A. M., M. D., Member of Philadelphia County Medical Society; Corresponding Member of the Gynæcological Society of Boston, etc. Philadelphia: George McLean.

The Cell Doctrine. Its History and Present state. For the use of Students in Medicine and Dentistry. By James Tyson, M. D., Lecturer on Microscopy, in the University of Pennsylvania, and on Physiology in the Pennsylvania College of Dental Surgery, etc. Colored Plate and Illustrations. Philadelphia: Lindsay and Blakiston. Received through T. Butler & Son.

Modern Therapeutics. A Compendium of Recent Formulas and Specific Therapeutical Directions. By George Napheys, A. M., M. D. One of the Editors of the Haly-Yearly Compendium of Medical Science; Chief of Medical Clinic of Jefferson Medical College, etc. Philadelphia. S. W. Butler, 115 South Seventh Street.

Relaxation of the Pelvic Symphyses during Pregnancy and Parturition. By Frederick G. Snellln[illegible], M. D. Reprinted from the American Journal of Obstetrics, February, 1870.

New Facts and Remarks concerning Idiocy. A Lecture delivered before the N. Y. City Medical Journal Association. By Edward Seguin, M. D.

Reports of the Trustees and Superintendent of the Butler Hospital for the Insane. Presented to the Corporation at their Annual Meeting, January 26th, 1870. Providence, R. I.

Constitution, By-Laws and List of Members of the Genesee County Medical Society, together with the code of ethics of the American Medical Association. Batavia, N. Y., 1869.

Report of the President Physician of Brigham Hall, a Hospital for the Insane, for the year 1869. Canandaigua, N. Y.

Review of Dr. Ruppaner's case of Laryngo-Tracheotomy. By Lewis A. Sayre, M. D.

